# Mosaic evolution of the skeleton in a clade of miniaturized turtles

**DOI:** 10.1098/rsbl.2026.0366

**Published:** 2026-11-04

**Authors:** Gerardo A. Cordero, John B. Iverson

**Affiliations:** ^1^ Cavanilles Institute of Biodiversity and Evolutionary Biology, University of Valencia, Paterna, Spain; ^2^ Department of Biology, Earlham College, Richmond, IN, USA

**Keywords:** body size evolution, dwarfism, miniaturization, skeletogenesis, shell evolution, megacephaly, osteology

## Abstract

Extreme body size reduction (miniaturization) is associated with the mosaic (trait-specific) evolution of the skeleton in vertebrate animals, though the relevant ecological context wherein shifts in osseous tissue production occur is challenging to evaluate. To address this concern, we examined skeletal mass variation in the most species-rich clade of freshwater turtles, comprised of miniaturized lineages that acquired the capacity to survive extended periods of time on land. The hypothesis that increasingly terrestrial behaviour would favour a relatively heavier skeleton was supported. Structural reinforcement of the postcranial skeleton in semi-terrestrial species featured the relative enlargement of the ventral shell and the refinement of functionally novel articulations that aid in protection from terrestrial predators and the environment. Furthermore, the spatial distribution of skeletal mass was consistent with a mosaic evolution trend wherein many aquatic species forego the protective benefits of a structurally robust shell and instead invest on much heavier skulls. This altered trait correlation points to a potential reallocation of osseous tissue from the cranial to the postcranial skeleton, which is probably linked to adaptation to terrestrial habitats in miniaturized turtles. Our study provides impetus to further clarify how skeletal traits are modified in the mosaic evolution of extremely small animals.

## 1. Introduction

Extreme body size reduction (miniaturization) is often linked to the trait-specific (mosaic) evolution of the skeleton in diverse vertebrate animals [1–3]. Miniaturized species may display unusual phenotypic correlations owing to uneven rates of growth and skeletal development [4,5]. Rates of skeletal ossification might be accelerated compared with the ancestral large-bodied condition [6]. For example, advanced cranial ossification is related to regionalized modifications, including novel structural rearrangements, within the skeleton of miniaturized amphibians [4,6–9]. Such developmental differences indicate that skeletal mass is not uniformly distributed and may evolve independently from body size. At the macroevolutionary scale, this assumption has not been explicitly tested within the context of miniaturization. In general, skeletal mass variation in vertebrates is rarely directly measured and compared across multiple species (but see [10]).

Miniaturization of the skeleton might influence adaptation to novel ecological niches [1–3,11,12]. In the aquatic stem (ancestral) lineages that gave rise to the most speciose clade of miniaturized turtles (Kinosternidae), a miniaturization event in the late Cretaceous was later followed by terrestrial niche expansion [13–15] (figure 1a). It was hypothesized that this freshwater clade is paedomorphic (juvenile-like) and, thus, species should exhibit incompletely ossified shells [17], yet we previously showed that embryonic ossification rates in species that acquired the capacity to survive extended periods of land (semi-terrestriality) are accelerated relative to a large-bodied outgroup lineage [18] (figure 1a). Whether such a heterochronic shift is ecologically relevant is unclear, given that aquatic kinosternids examined thus far also exhibit hyperossification [18].

**Figure 1.**
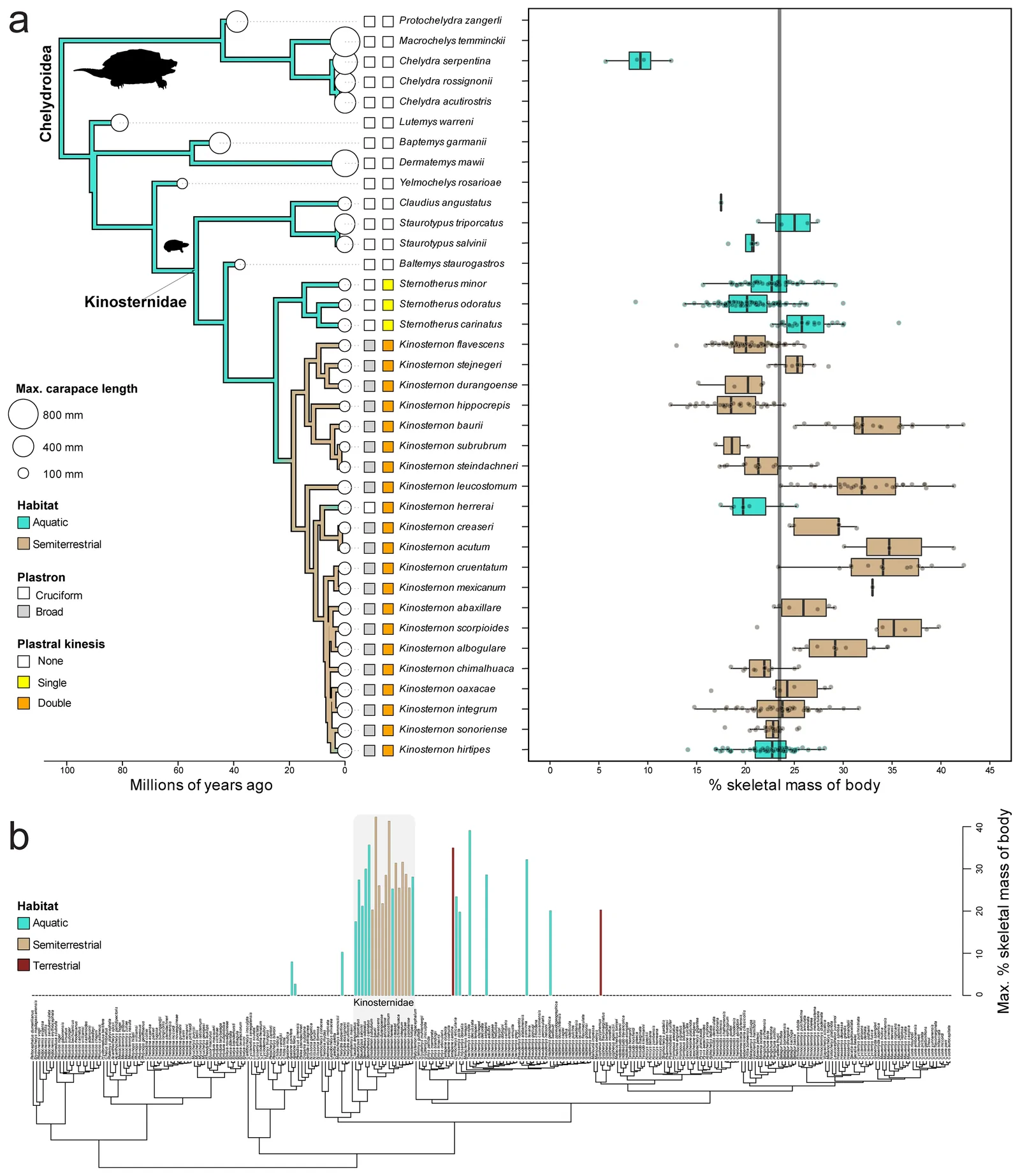
(a) On the basis of the maximum CL recorded for species, a phyletic miniaturization trend is inferred across the *Chelydroidea* superfamily of freshwater turtles. Reconstructed ancestral states for habitat suggest that semi-terrestriality co-evolved with ventral shell (plastron) morphology and mobility (kinesis) in small-bodied species. Silhouettes were accessed through https://www.phylopic.org via Creative Commons licenses. (a, right panel) Many semi-terrestrial species feature greater skeletal mass relative to body mass (estimated by direct measurement or predicted via regression; *n* = 524). The vertical line denotes the grand mean for relative skeletal mass in *Kinosternidae* by species, whereas box plots denote species’ medians and interquartile ranges. (b) Presently available estimates for maximum relative skeletal mass were mapped onto the phylogeny of Thomson *et al.* [16]. Habitat preference for species for which relative skeletal mass has been estimated is also highlighted.

In vertebrate animals, terrestrial and shallow-water environments may select for dense skeletons to support terrestrial locomotion and buoyancy, respectively [19,20]. In turtles, terrestrial environments are assumed to select for heavier skeletons that minimize desiccation and enhance anti-predatory defence [21–23]. Even so, as in most vertebrates, skeletal mass data are scarce in the literature. The presently available estimates of whole skeletal mass in relation to body mass suggest that semi-terrestrial kinosternids are comparable with much larger freshwater turtles representing other clades [24–26]. These estimates on their own, however, do not inform how the mass of distinct skeletal modules evolved. The distribution of skeletal mass within the body may differ, perhaps owing to adaptive variation in skull size and shell morphology [27,28]. Aquatic kinosternids display megacephaly and a relatively small ventral shell (plastron) [27]. By contrast, semi-terrestrial species display a box turtle phenotype characterized by plastral enlargement, as observed across the turtle tree of life in some small-bodied terrestrial species [27,29].

To clarify how skeletal mass variation evolves within a relevant ecological and macroevolutionary context, the present paper aimed to: (i) Test the hypothesis that semi-terrestrial miniaturized kinosternid turtles feature heavier skeletons compared with aquatic species. (ii) Test whether aquatic-to-land transitions influenced the mosaic evolution of skeletal trait modules, possibly via shifts in the allocation of osseus tissue production during ontogeny. Mosaic evolution was evaluated by comparing interspecific differences in the scaling of skeletal mass components in reproductively mature individuals, that is evolutionary allometry [30,31].

## 2. Material and methods

### (a) Specimen processing and classification

Museum specimens collected in taxonomic and life history studies by Iverson and colleagues [25,32–35] were examined. The specimens were deposited in the Florida State Museum (UF). Maximum straight-line measurements of carapace length (CL) and plastron length (PL) were recorded (with calipers) at the midline to the nearest 0.2 mm. Sex was assigned by observing secondary sexual characters that are well characterized in the Kinosternidae: Differences in tail length, relative position of the cloacal opening, and male-specific clasping organs [36,37]. Sex assignment was often validated by direct observation of reproductive organs during dissection. About 591 reproductively mature specimens were retained for analyses after excluding juveniles and damaged specimens, or parts thereof. It was not possible to determine the sex of 12 individuals, though they were assumed to be reproductively mature based on CL or mass (see supplementary material). Theory predicts that reptiles near 70% of maximal adult body size should be reproductively mature [38]. However, our analyses included turtles with secondary sexual characters that did not approach 70% of the maximum CL observed for a given species. This was expected because age of maturity and body size may vary geographically, and our specimens often originated from multiple localities.

### (b) Skeletal and body mass estimates

Carcasses were macerated in water at room temperature. Following at least six months of air-drying, skeletons were weighed to the nearest 0.1 g. Scutes and cartilages were mostly detached and degraded after maceration and drying. Total skeletal mass, shell mass (plastron + carapace with attached vertebrae), and skull mass (excluding mandible) were recorded. Postcranial mass was the difference between skull (cranial) mass and total skeletal mass. Appendicular mass was also estimated by subtracting the sum of cranial and shell mass from the total skeletal mass. Live body mass could only be measured in 206 specimens. In 318 unweighed specimens, body mass was predicted via species-specific regressions of CL and body mass extracted from a turtle life history database [39]. Species’ means and sample sizes for all skeletal traits measured are reported in supplementary material, table S1. The final dataset, including additional PL and CL data (*n* = 38) sampled by Cordero [40], is provided in the supplementary material.

### (c) Habitat preference categorization

Kinosternid turtles are primarily freshwater turtles typically found in shallow depths. The genera *Claudius*, *Staurotypus* and *Sternotherus* are unequivocally aquatic and exclusively utilize terrestrial habitats for egg-laying. The genus *Kinosternon* is exceptional in that, other than for egg-laying, it has evolved the capacity to withstand lengthy stays on land. For the purposes of this study, we categorize this capacity as ‘semi-terrestrial’, though it should be noted that this genus might be better described as one that occupies a dynamic aquatic-terrestrial ecological niche spectrum. In any case, our operational definition is supported by natural history accounts and our own field observations validating long-distance terrestrial travel during droughts, displacement between wetlands, and overwintering or estivation in terrestrial burrows [41–43]. These behaviours are not consistently displayed by *K. herrerai* and *K. hirtipes*, which are generally accepted as the only exclusively aquatic members of *Kinosternon* [41].

### (d) Comparative phylogenetic framework

On the basis of morphology [27], fossils [44] and previous comparative phylogenetic analyses [18,45], the hypothesis that *Kinosternon* is derived from a strictly aquatic ancestor is not disputed. Nonetheless, we provide an updated ancestral state reconstruction of habitat preference to illustrate the comparative framework guiding the interpretation of our results. Using the *ace* function of the *ape* R package [46], maximum likelihood ancestral state reconstruction of habitat preference (assuming an equal rates evolutionary model) was performed on the most recent phylogeny for Kinosternidae [47], with outgroup lineages (Chelydroidea: Chelydridae + Dermatemydidae) grafted from the most recent molecular phylogeny of Testudines [16]. This phylogeny had been previously time-calibrated, following the guidelines of Joyce *et al.* [48]. Well-described fossils representing the earliest-branching lineages within Chelydroidea were grafted along branches based on their estimated ages [13,15,49]. Using the *make.simmap* and *plotTree* functions of *phytools* [50], 1000 evolutionary histories for habitat preference were simulated along branches and overlaid onto the phylogeny to visualize habitat preference transitions.

Potential sources of skeletal mass variation related to habitat preference include plastron shape (cruciform or broad) and the presence/absence and type of plastral kinesis. These are diagnostic characters commonly used to assign habitat preference to fossil turtles [51]. Character states were mapped onto tips of the phylogeny together with distributions for relative skeletal mass. Moreover, the phyletic miniaturization trend previously described in Chelydroidea, see [18], was summarized by plotting points proportional to maximal CL onto tips. Maximal CL for Kinosternidae is based on the present dataset, whereas values for Chelydroidea outgroups were from the literature, as detailed in Cordero [18]. Fossil CL was estimated by Holroyd *et al.* [15]. To ascertain whether trends in Chelydroidea are consistent across turtle phylogeny, maximum values for skeletal mass relative to body mass were plotted using data from Iverson [25].

### (e) Statistical assessment of mosaic evolution

Evolutionary allometry regression models enable inference on how skeletal traits change relative to one another in different species. When sampling specimens at or approaching reproductive maturity, it is assumed that shifts in the allometric intercepts of regression models are the product of divergent ontogenetic trajectories under different selective regimes [30,31,52]. As such, we tested three evolutionary allometry hypotheses: (i) The plastron undergoes relative enlargement owing to the ‘box turtle’ effect in semi-terrestrial species [25,29,51], resulting in a heavier shell and postcranial skeleton; (ii) Megacephaly is mainly restricted to aquatic species [27], indicating a redistribution of osseous tissue from the cranial to postcranial skeleton [27]. (iii) The appendicular skeleton is heavier to support locomotion and digging on land [53].

Hypotheses were tested via phylogenetically corrected generalized linear models (PGLS) models on shell mass/PL-to-CL ratio, PL/CL, cranial mass/shell mass, cranial mass/PL-to-CL ratio, and appendicular mass/shell mass. Differences in allometric coefficients were tested with habitat as the predictor. Models were constructed with the *gls* function of *nlme*, with phylogenetic covariance structures specified via *ape*. Using the *phylosig* function of *phytools*, the phylogenetic signal was estimated to be moderate in residuals derived from ordinary least squares regression (Blomberg's *K* = 0.19–0.39). It was determined via likelihood ratio tests that the Ornstein–Uhlenbeck covariance structure was better fitted to PGLS models on cranial mass, whereas the Brownian motion structure was supported in other models.

Incomplete sampling precluded the inclusion of sex as a factor in models. However, distributions grouped by sex for cranial mass, postcranial mass, and relative size of the plastron (the PL-to-CL ratio) were graphically compared. Kinesis type and cruciform/broad shell states were also excluded from the PGLS analyses owing to high collinearity with shell mass and the PL-to-CL ratio. Moreover, species’ means for shell mass relative to body mass and total skeletal mass, and the PL-to-CL ratio were plotted. Cranial mass was plotted against postcranial mass to illustrate the striking megacephaly (exaggerated skull size) in aquatic kinosternids [27].

## 3. Results

### (a) Heavier skeletons in semi-terrestrial species

An aquatic ancestral state (posterior probabilities: aquatic = 0.997, semi-terrestrial = 0.002) and two recent reversals from semi-terrestrial to aquatic habitat preference were supported in the Kinosternidae (figure 1a). The transition to terrestriality probably followed the divergence of *Sternotherus* and *Kinosternon*, whose shells are defined by cruciform and broad plastral morphologies, respectively (posterior probabilities: aquatic = 0.846, semi-terrestrial = 0.153; figure 1a). Semi-terrestrial species tended to display greater proportional skeletal mass (figure 1a; supplementary material, table S1), with maximal values near or above 40% of body mass. These estimates ranged from 5.7% to 12.4% in an aquatic large-bodied representative of the outgroup *Chelydroidea* lineage (see *Chelydra serpentina*; figure 1a). However, across turtle phylogeny, habitat preference was not seemingly associated with maximal skeletal mass as a percentage of body mass (figure 1b).

Proportional shell mass values were greater in semi-terrestrial species (figure 2a,b). The PL-to-CL ratio was highest in semi-terrestrial species, particularly in females (figure 2c,d). Although differences in intercepts for the shell mass/PL-to-CL ratio relationship were not supported with high statistical confidence (table 1), the allometric intercept for the PL/CL relationship was greatest in semi-terrestrial species (figure 2e; table 1). The resulting differences in aquatic (ancestral cruciform plastron) versus semi-terrestrial (derived broad plastron) lineages can be qualitatively discerned by inspection of plastral size and shape relative to the carapace (figure 2f,g).

**Figure 2.**
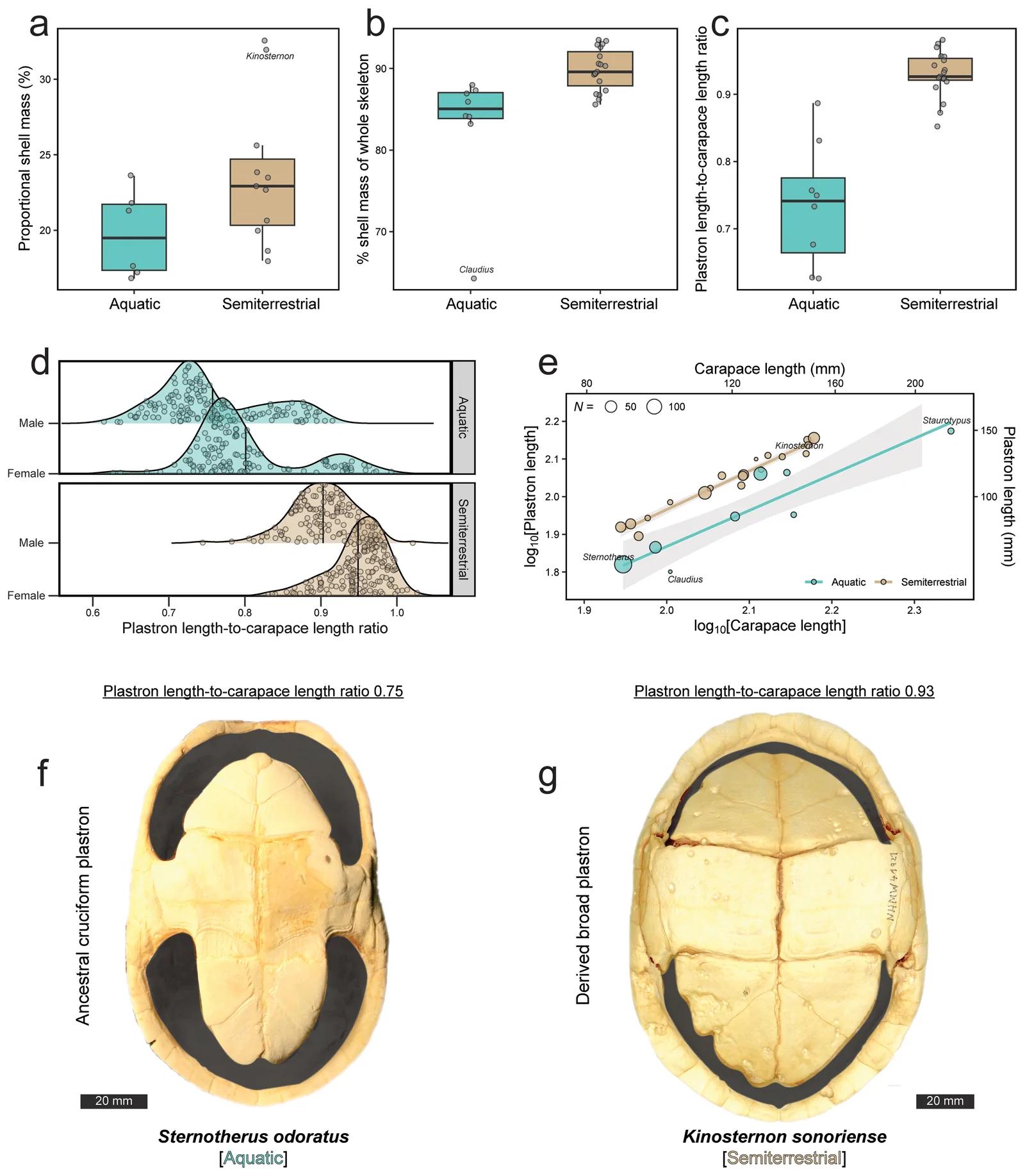
(a–c) Proportional shell mass, shell mass relative to total skeletal mass and the CL-to-PL ratio tend to be higher in semi-terrestrial species. Box plots depict medians and interquartile ranges of habitat groups. (d) The CL-to-PL ratio is generally higher in semi-terrestrial females. Circles represent reproductively mature individuals and lines denote the means of distributions. (e) The PL/CL relationship differed according to the habitat group. Fitted lines are based on ordinary least squares regression. (f,g) Differences in shell mass and the allometric intercept for the PL and CL relationship are qualitatively evident in skeletonized shells of aquatic versus semi-terrestrial representative species. *Kinonsternon sonoriense* (NHMW41921) exhibits a box turtle phenotype.

**Table 1.** Results of phylogenetic generalized least squares models on skeletal traits of miniaturized kinosternid turtles (*n* = 27 species).

response [log_10_]	predictors	estimates	CI	*p*
shell mass	intercept	1.24	−0.30 to 2.78	0.109
	PL-to-CL ratio	0.66	−1.41 to 2.74	0.515
	habitat	−0.05	−0.52 to 0.42	0.820
PL	intercept	−0.27	−0.55 to −0.01	0.049
	CL [log_10_]	1.06	0.94–1.19	<0.001
	habitat	0.04	0.01–0.07	0.009
shell mass	intercept	1.23	1.05–1.40	<0.001
	cranial mass [log_10_]	0.93	0.68–1.19	<0.001
	habitat	0.34	0.18–0.49	<0.001
cranial mass	intercept	1.36	0.00–2.71	0.049
	PL-to-CL ratio	−1.11	−2.93 to 0.71	0.221
	habitat	−0.06	−0.48 to 0.35	0.753
appendicular mass	Intercept	−0.51	−1.09 to 0.07	0.082
	shell mass[log_10_]	0.81	0.58–1.04	<0.001
	habitat	−0.12	−0.30 to 0.06	0.178

### (b) Allometric shifts support mosaic evolution

The postcranial skeleton of semi-terrestrial females was the heaviest (figure 3a), whereas the cranial skeleton tended to be heavier in aquatic males (figure 3b). In absolute and relative terms, the heaviest and largest cranial skeletons belonged to the earliest-branching aquatic kinosternids (figure 3c,d). The skull is nominally much smaller relative to the shell in the *Kinosternon* semi-terrestrial subclade, which exhibits a box turtle configuration wherein the plastron is elevated via movable hinge articulations (figure 3d). This was consistent with an elevated intercept in the shell mass/cranial mass allometric relationship (figure 3e; table 1). Intercepts did not differ considerably in the cranial mass/PL-to-CL ratio and appendicular mass/shell mass models (figure 3f,g; table 1).

**Figure 3.**
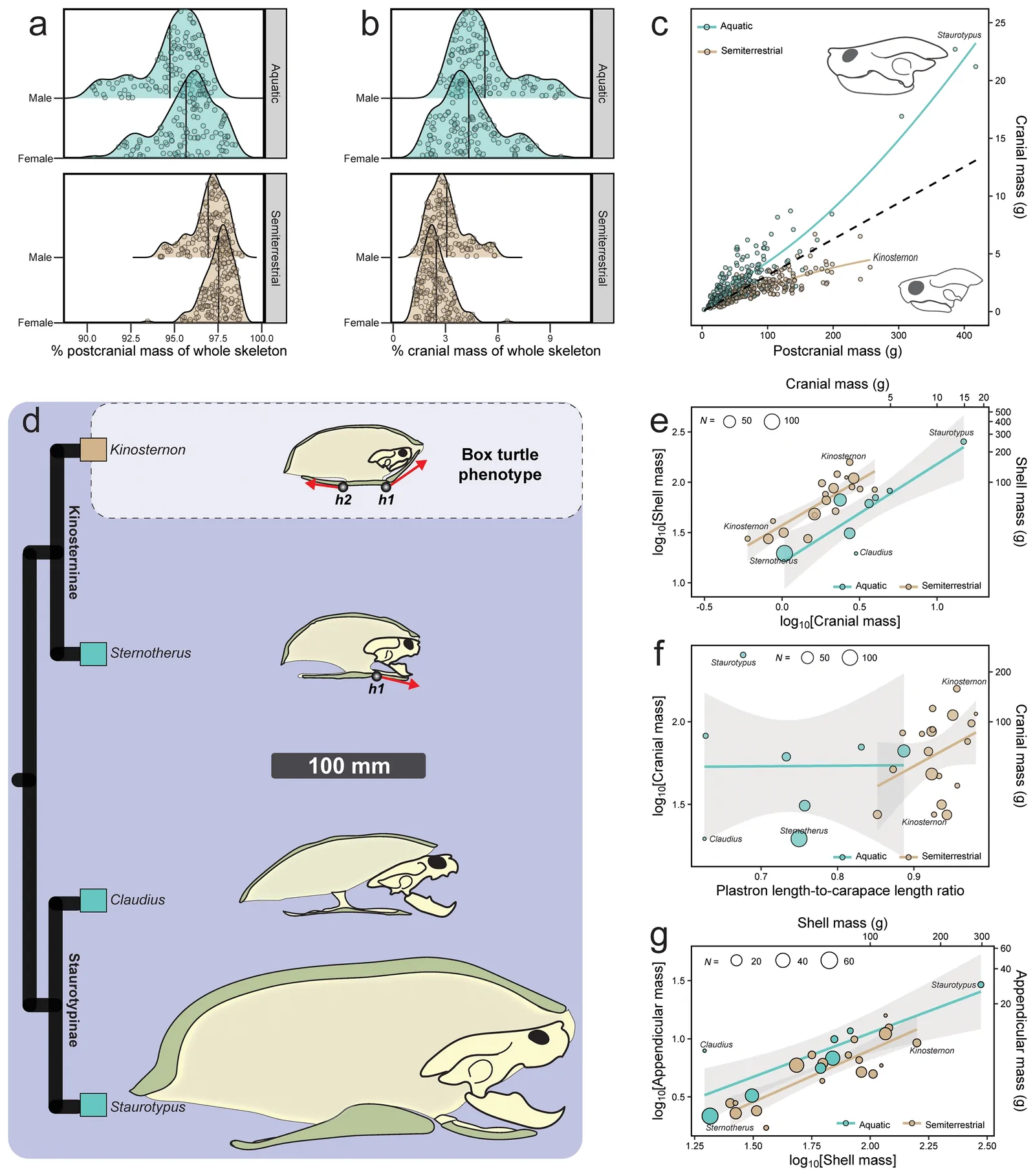
(a,b) Postcranial skeletal mass relative to whole skeletal mass tended to be higher in semi-terrestrial females, whereas relative cranial mass was higher in aquatic males. Circles represent reproductive individuals and vertical lines denote the means of distributions. (c) Cranial mass plotted against postcranial mass with the moving average fitted according to habitat. Circles represent reproductive individuals, whereas the dashed line indicates isometry. (d) Plastron morphology is associated with skull size and kinesis. Hinges (*h1-h2*) are shown with red arrows denoting the direction of movement. Illustrations were modified from Bramble *et al.* [27]. (e) The shell mass/cranial mass relationship in aquatic versus semi-terrestrial species features similar slopes but with dissimilar intercepts. (f) Aquatic and semi-terrestrial species share similar allometric coefficients but with dissimilar offsets in the relationship between cranial mass and the PL-to-CL ratio. (g) The allometric intercept did not differ markedly when regressing appendicular mass against shell mass. Circles represent species’ means, and fitted lines are based on ordinary least squares regression.

## 4. Discussion

We conducted one of the most exhaustive comparative surveys of reptilian skeletal mass variation to date and provided evidence of mosaic skeletal evolution in semi-terrestrial lineages of the most species-rich clade of miniaturized turtles. Our findings suggest that the production and spatial distribution of osseous tissue are altered in the shell and skull of semi-terrestrial species, some of which feature the heaviest skeletons in proportion to body mass thus far recorded in turtles.

### (a) Terrestriality may select for heavier skeletons

Transitions to terrestriality in vertebrate evolution involve increases in supportive skeletal tissue to sustain locomotion without the buoyant effect of water [20]. Such signatures of habitat-associated selection are well recognized in turtles [54–56]. Although heavy skeletons are common in terrestrial species with structurally reinforced shells [25,57], skeletal mass may be gained via selection for enhanced buoyancy control in shallow water habitats [19]. The Kinosternidae are primarily bottom-walking species that inhabit shallow-water habitats. Thus, selection for heavier skeletons might be collectively magnified by both functional demands in shallow water and on land in semi-terrestrial species. In either case, our analyses support an evolutionary scenario wherein ecological niche expansion into terrestrial habitats coincided with heavier skeletons in the Kinosternidae. The shell and postcranial skeleton tended to be proportionally heavier in semi-terrestrial species that acquired two kinetic hinges and broad plastra that deviate from the cruciform morphology of aquatic species. This suite of skeletal modifications is probably adaptive, as it enhances anti-predatory defence and minimizes dehydration by reducing exposed skin surface area [22,29]. As such, the plastron tended to be relatively larger in females that must venture onto land to construct nests.

Skeletal mass is on average about 20%–28% of total body mass in turtles [25,26], which is expected because heavy osseous tissue is the predominant component of their shelled bodies. These values are among the highest that have thus been reported in vertebrates, though data for other armoured taxa, to the best of our knowledge, are unavailable. Perhaps more remarkable is that the skeletal mass/body mass scaling relationship was augmented in some miniaturized semi-terrestrial kinosternids. We documented some of the highest values for proportional skeletal mass ever reported for turtles, which might be explained by the decoupling of growth and skeletal maturation rates. This is evident in modular alterations, including the incomplete ossification of the cartilage-derived scapula [58], as well as the reorganization, numerical reduction, and hyperossification of dermis-derived (shell) bones [18,59]. Such within-organism differences are hallmarks of mosaic evolution in miniaturized vertebrate animals [4,6].

### (b) Mosaic evolution of skeletal mass components

Shifts in skeletal mass allometries imply that energetic resources for osseous tissue production are distributed unevenly in development, thereby contributing to mosaic changes within the skeleton. Our evolutionary allometry analyses suggest that semi-terrestrial kinosternids have redirected osseous mass from the cranial to the postcranial skeleton, yielding relatively smaller skulls that reflect an increasingly omnivorous diet while on land [27]. Smaller skulls also enable withdrawal into the shell, though such a ‘box turtle’ effect did not co-occur with reduced appendicular mass. The allometry of body and limb size remains highly evolutionarily conserved in turtles, possibly owing to the presence of the shell [60]. Still, the acquisition of an additional hinge articulation in semi-terrestrial *Kinosternon* renders the shell a formidable defensive structure, as in other small-bodied terrestrial clades of Testudines [29,45].

Aquatic kinosternids allocate more osseous tissue to the skull, resulting in megacephaly to support a diet of hard-shelled mollusks and crustaceans [26]. These species invest less energy in shell development, as reflected by a cruciform plastron with a single or no hinge. Following reversals to the ancestral aquatic preference, the reacquisition of a cruciform plastron and structural weakening of the second plastral hinge in some *Kinosternon* species underscore the capacity for the skeleton to evolve in a modular manner. Such differences in skeletal mass allometries probably correspond to interspecific variation in the density, size, and shape of adult bones. Modifying these structural properties is not trivial because it requires the alteration of cell proliferation and division, coupled with remodelling of the extracellular matrix during embryonic and juvenile life stages [61]. Thus, additional research on skeletal development and maturation would ideally validate mosaic evolution hypotheses based on evolutionary allometry models.

### (c) Broader phylogenetic implications and conclusions

It is challenging to extrapolate our findings to other turtle taxa, as demonstrated by our comparison of the scarcely available data in the literature. Nevertheless, considering that the alignment of ecological and phenotypic trends across turtle phylogeny is often noisy and context-dependent [56,62,63], our study on the miniaturized kinosternid clade should motivate further questions on how mosaic skeletal changes track the diversification of turtle species. More broadly, our study invites further inquiry into how skeletal mass variation evolves in diverse vertebrate animals, with the aim of clarifying the extent to which spatial constraints in development and environmental selective pressures jointly influence the modularity of the skeleton.

## Supplementary Material



## Data Availability

Data are available in the supplementary material [64].

## References

[1] Kammerer CF , Nesbitt SJ, Flynn JJ, Ranivoharimanana L, Wyss AR. 2020 A tiny ornithodiran archosaur from the Triassic of Madagascar and the role of miniaturization in dinosaur and pterosaur ancestry. Proc. Natl Acad. Sci. USA 117, 17 932–17 936. (doi:10.1073/pnas.1916631117) PMC7395432 32631980

[2] Lautenschlager S , Gill PG, Luo ZX, Fagan MJ, Rayfield EJ. 2018 The role of miniaturization in the evolution of the mammalian jaw and middle ear. Nature 561, 533–537. (doi:10.1038/s41586-018-0521-4) 30224748

[3] Lee MS , Cau A, Naish D, Dyke GJ. 2014 Dinosaur evolution. Sustained miniaturization and anatomical innovation in the dinosaurian ancestors of birds. Science 345, 562–566. (doi:10.1126/science.1252243) 25082702

[4] Hanken J , Wake DB. 1993 Miniaturization of body size: organismal consequences and evolutionary significance. Annu. Rev. Ecol. Syst. 24, 501–519. (doi:10.1146/annurev.es.24.110193.002441)

[5] Dobreva MP , Camacho J, Abzhanov A. 2022 Time to synchronize our clocks: connecting developmental mechanisms and evolutionary consequences of heterochrony. J. Exp. Zool. B Mol. Dev. Evol. 338, 87–106. (doi:10.1002/jez.b.23103) 34826199

[6] Hanken J . 1993Adaptation of bone growth to miniaturization of body size. In Bone, volume 7: bone growth—B (ed. B Hall), pp. 79–104. Boca Raton, FL: CRC Press.

[7] Hanken J . 1985 Morphological novelty in the limb skeleton accompanies miniaturization in salamanders. Science 229, 871–874. (doi:10.1126/science.4023715) 4023715

[8] Clemente‐Carvalho RBG *et al.* 2009 Hyperossification in miniaturized toadlets of the genus *Brachycephalus* (Amphibia: Anura: Brachycephalidae): microscopic structure and macroscopic patterns of variation. J. Morphol. 270, 1285–1295. (doi:10.1002/jmor.10755) 19551869

[9] Pérez-Ben CM , Schoch RR, Báez AM. 2018 Miniaturization and morphological evolution in Paleozoic relatives of living amphibians: a quantitative approach. Paleobiology 44, 58–75. (doi:10.1017/pab.2017.22)

[10] Martin-Silverstone E , Vincze O, McCann R, Jonsson CH, Palmer C, Kaiser G, Dyke G. 2015 Exploring the relationship between skeletal mass and total body mass in birds. PLoS ONE 10, e0141794. (doi:10.1371/journal.pone.0141794) PMC4625084 26509531

[11] Paluh DJ , Stanley EL, Blackburn DC. 2020 Evolution of hyperossification expands skull diversity in frogs. Proc. Natl Acad. Sci. USA 117, 8554–8562. (doi:10.1073/pnas.2000872117) PMC7165440 32220958

[12] Rensch B . 1948 Histological changes correlated with evolutionary changes of body size. Evolution 2, 218–230. (doi:10.1111/j.1558-5646.1948.tb02742.x) 18884663

[13] Joyce WG , Bourque JR. 2016 A review of the fossil record of turtles of the clade Pan-Kinosternoidea. Bull. Peabody Mus. Nat. Hist. 57, 57–95. (doi:10.3374/014.057.0104)

[14] Joyce WG , Claude J. 2020 An alternative interpretation of the Paleogene turtle *Cardichelyon rogerwoodi* as a hinged kinosternoid. J. Paleontol. 94, 557–567. (doi:10.1017/jpa.2019.92)

[15] Holroyd PA , Hutchison JH, Strait SG. 2001Turtle diversity and abundance through the lower Eocene Willwood formation of the southern Bighorn basin. In Paleocene-Eocene stratigraphy and biotic change in the Bighorn and Clarks Fork Basins, Wyoming, University of Michigan Papers on Paleontology (ed. PD Gingerich), vol. 33, pp. 97–107. Ann Arbor, MI: University of Michigan.

[16] Thomson RC , Spinks PQ, Shaffer HB. 2021 A global phylogeny of turtles reveals a burst of climate-associated diversification on continental margins. Proc. Natl Acad. Sci. USA 118, e2012215118. (doi:10.1073/pnas.2012215118) PMC7896334 33558231

[17] Heston L , Meylan P, Goessling JM. 2022 Life history consequences of miniaturization in turtles: evidence from the subfamily *Kinosterninae* (Testudines: Kinosternidae). Biol. J. Linn. Soc. 135, 558–568. (doi:10.1093/biolinnean/blab163)

[18] Cordero GA . 2021 Disentangling the correlated evolution of body size, life history and ontogeny in miniaturized chelydroid turtles. Evol. Dev. 23, 439–458. (doi:10.1111/ede.12386) 34037309

[19] Houssaye A . 2009 ‘Pachyostosis’ in aquatic amniotes: a review. Integr. Zool. 4, 325–340. (doi:10.1111/j.1749-4877.2009.00146.x) 21392306

[20] Reynolds WW . 1977 Skeleton weight allometry in aquatic and terrestrial vertebrates. Hydrobiologia 56, 35–37. (doi:10.1007/BF00023283)

[21] Pritchard PCH . 2008Evolution and structure of the turtle shell. In Biology of turtles (eds J Wyneken, MH Godfrey, V Bels), pp. 45–84. Boca Raton, FL: CRC Press.

[22] Stone PA , Iverson JB. 1999 Cutaneous surface area in freshwater turtles. Chelonian Conserv. Biol. 3, 512–515.

[23] Cherepanov G . 2019 Morphogenetic and constructional differences of the carapace of aquatic and terrestrial turtles and their evolutionary significance. J. Morphol. 280, 1571–1581. (doi:10.1002/jmor.21050) 31411770

[24] Prange HD , Anderson JF, Rahn H. 1979 Scaling of skeletal mass to body mass in birds and mammals. Am. Nat. 113, 103–122. (doi:10.1086/283367)

[25] Iverson JB . 1984 Proportional skeletal mass in turtles. Fla. Sci. 47, 1–11.

[26] Sterrett SC , Maerz JC, Katz RA. 2015 What can turtles teach us about the theory of ecological stoichiometry? Freshw. Biol. 60, 443–455. (doi:10.1111/fwb.12516)

[27] Bramble DM , Hutchison JH, Legler JM. 1984 Kinosternid shell kinesis: structure, function and evolution. Copeia 1984, 456. (doi:10.2307/1445203)

[28] Berlant ZS , Stayton TC. 2017 Shell morphology in the Kinosternidae: functional and evolutionary patterns. Herpetologica 73, 30. (doi:10.1655/herpetologica-d-15-00064)

[29] Cordero GA . 2023 Turtle shell kinesis underscores constraints and opportunities in the evolution of the vertebrate musculoskeletal system. Integr. Org. Biol. 5, obad033. (doi:10.1093/iob/obad033) PMC10576247 37840690

[30] Cheverud JM . 1982 Relationships among ontogenetic, static and evolutionary allometry. Am. J. Phys. Anthropol. 59, 139–149. (doi:10.1002/ajpa.1330590204) 7149015

[31] Klingenberg CP . 1998 Heterochrony and allometry: the analysis of evolutionary change in ontogeny. Biol. Rev. 73, 79–123. (doi:10.1111/j.1469-185X.1997.tb00026.x) 9569772

[32] Iverson JB . 1991 Phylogenetic hypotheses for the evolution of modern kinosternine turtles. Herpetol. Monogr. 5, 1. (doi:10.2307/1466974)

[33] Iverson JB , Balgooyen CP, Byrd KK, Lyddan KK. 1993 Latitudinal variation in egg and clutch size in turtles. Can. J. Zool. 71, 2448–2461. (doi:10.1139/z93-341)

[34] Iverson JB , Le M, Ingram C. 2013 Molecular phylogenetics of the mud and musk turtle family Kinosternidae. Mol. Phylogenet. Evol. 69, 929–939. (doi:10.1016/j.ympev.2013.06.011) 23823161

[35] Macip-Ríos R , Ontiveros RN, Sánchez-León A, Casas-Andreu G. 2013 Evolution of reproductive effort in mud turtles (Kinosternidae): the role of environmental predictability. Evol. Ecol. Res. 18, 539–554.

[36] Ceballos CP , Iverson JB. 2014 Patterns of sexual size dimorphism in Chelonia: revisiting Kinosternidae. Biol. J. Linn. Soc. 111, 806–809. (doi:10.1111/bij.12273)

[37] López-González NE , López-Luna MA, Escobedo-Galván AH. 2025 Sexual size dimorphism and allometric growth of the smallest turtle in the world (Testudines: Kinosternidae). Salamandra 61, 95–100.

[38] Shine R , Iverson JB. 1995 Patterns of survival, growth and maturation in turtles. Oikos 72, 343. (doi:10.2307/3546119)

[39] Iverson JB , Ennen J, Lovich J. 2025 Life-history and ecology data for the turtles of the world. Chelonian Conserv. Biol. 24, 136–141. (doi:10.2744/ccb-1646.1)

[40] Cordero GA . 2018 Is the pelvis sexually dimorphic in turtles? Anat. Rec. 301, 1382–1389. (doi:10.1002/ar.23831) 29677702

[41] Legler JM , Vogt RC. 2013 The turtles of Mexico: land and freshwater forms. Berkeley, NC: University of California Press. (doi:10.1525/9780520956896)

[42] Ernst CH , Lovich JE. 2009 Turtles of the United States and Canada. Baltimore, MD: Johns Hopkins University Press. (doi:10.56021/9780801891212)

[43] Cordero GA , Swarth CW. 2010 Notes on the movement and aquatic behavior of some kinosternid turtles. Acta Zool. Mex. 26, 233–235. (doi:10.21829/azm.2010.261692)

[44] Bourque JR . 2016 New mud turtles (Kinosternidae, *Kinosternon*) from the middle–late Miocene of the United States. J. Paleontol. 89, 821–844. (doi:10.1017/jpa.2015.63)

[45] Cordero GA , Quinteros K, Janzen FJ. 2018 Delayed trait development and the convergent evolution of shell kinesis in turtles. Proc. R. Soc. B 285, 20181585. (doi:10.1098/rspb.2018.1585) PMC6191694 30282655

[46] Paradis E , Schliep K. 2019 ape 5.0: an environment for modern phylogenetics and evolutionary analyses in R. Bioinformatics 35, 526–528. (doi:10.1093/bioinformatics/bty633) 30016406

[47] Hurtado-Gómez JP , Vargas-Ramírez M, Iverson JB, Joyce WG, McCranie JR, Paetzold C, Fritz U. 2024 Diversity and biogeography of South American mud turtles elucidated by multilocus DNA sequencing (Testudines: Kinosternidae). Mol. Phylogenet. Evol. 197, 108 083. (doi:10.1016/j.ympev.2024.108083) 38679303

[48] Joyce WG , Parham JF, Lyson TR, Warnock RCM, Donoghue PCJ. 2013 A divergence dating analysis of turtles using fossil calibrations: an example of best practices. J. Paleontol. 87, 612–634. (doi:10.1666/12-149)

[49] Joyce WG . 2016 A review of the fossil record of turtles of the clade Pan-Chelydridae. Bull. Peabody Mus. Nat. Hist. 57, 21–56. (doi:10.3374/014.057.0103)

[50] Revell LJ . 2024 phytools 2.0: an updated R ecosystem for phylogenetic comparative methods (and other things). PeerJ 12, e16505. (doi:10.7717/peerj.16505) PMC10773453 38192598

[51] Hutchison JH . 1991 Early kinosterninae (Reptilia: Testudines) and their phylogenetic significance. J. Vertebr. Paleontol. 11, 145–167. (doi:10.1080/02724634.1991.10011385)

[52] Tidière M , Lemaître J‐F, Pélabon C, Gimenez O, Gaillard J‐M. 2017 Evolutionary allometry reveals a shift in selection pressure on male horn size. J. Evol. Biol. 30, 1826–1835. (doi:10.1111/jeb.13142) 28703357

[53] Young VKH , Baeza JA, Blob RW. 2019 Comparative limb bone scaling in turtles: phylogenetic transitions with changes in functional demands? J. Morphol. 280, 593–603. (doi:10.1002/jmor.20968) 30811074

[54] Bhat MS , Cullen TM. 2025 Growth and life history of freshwater chelydrid turtles (Testudines: Cryptodira): a bone histological approach. J. Anat. 247, 518–541. (doi:10.1111/joa.14130) PMC12397064 39169639

[55] Claude J , Pritchard P, Tong H, Paradis E, Auffray JC. 2004 Ecological correlates and evolutionary divergence in the skull of turtles: a geometric morphometric assessment. Syst. Biol. 53, 933–948. (doi:10.1080/10635150490889498) 15764561

[56] Foth C , Rabi M, Joyce WG. 2016 Skull shape variation in extant and extinct Testudinata and its relation to habitat and feeding ecology. Acta Zool. 98, 310–325. (doi:10.1111/azo.12181)

[57] Miller K , Birchard GF. 2005 Influence of body size on shell mass in the ornate box turtle, *Terrapene ornata*. J. Herpetol. 39, 158–161. (doi:10.1670/0022-1511(2005)039[0158:IOBSOS]2.0.CO;2)

[58] Cordero GA , Quinteros K. 2015 Skeletal remodelling suggests the turtle's shell is not an evolutionary straitjacket. Biol. Lett. 11, 20 150 022. (doi:10.1098/rsbl.2015.0022) PMC4424616 25878046

[59] Cordero GA , Vlachos E. 2021 Reduction, reorganization and stasis in the evolution of turtle shell elements. Biol. J. Linn. Soc. 134, 892–911. (doi:10.1093/biolinnean/blab122)

[60] Hermanson G , Evers SW. 2024 Shell constraints on evolutionary body size-limb size allometry can explain morphological conservatism in the turtle body plan. Ecol. Evol. 14, e70504. (doi:10.1002/ece3.70504) PMC11557996 39539674

[61] Hall BK . 2014 Bones and cartilage: developmental and evolutionary skeletal biology. San Diego: Elsevier Science. (doi:10.1016/C2013-0-00143-0)

[62] Hermanson G , Benson RBJ, Farina BM, Ferreira GS, Langer MC, Evers SW. 2022 Cranial ecomorphology of turtles and neck retraction as a possible trigger of ecological diversification. Evolution 76, 2566–2586. (doi:10.1111/evo.14629) PMC9828723 36117268

[63] Farina BM , Godoy PL, Benson RBJ, Langer MC, Ferreira GS. 2023 Turtle body size evolution is determined by lineage-specific specializations rather than global trends. Ecol. Evol. 13, e10201. (doi:10.1002/ece3.10201) PMC10293707 37384241

[64] Cordero GA , Iverson JB. 2026 Supplementary material from: Mosaic evolution of the skeleton in a clade of miniaturized turtles. Figshare (doi:10.6084/m9.figshare.c.8745901) PMC13730805 42976946

