# Supplementary material for: Mosaic evolution of the skeleton in a clade of miniaturized turtles
Source: Biol Lett. 2026 Nov 4;22(11):20260366. doi: 10.1098/rsbl.2026.0366 (PMC13730805; doi:10.1098/rsbl.2026.0366)
Supplement: Supplementary file 1 [file rsbl.2026.0366.zip › rsbl.2026.0366/rsbl20260366_si_001.pdf]

SUPPLEMENTARY ONLINE MATERIAL

Mosaic evolution of the skeleton in a clade of miniaturized turtles

Cordero, G.A. | Iverson, J.

Supplementary Table 1. Species' means for shell dimensions, skeletal mass measurements, and body mass are given. Also shown are means for plastron length (PL), carapace length (CL) and the PL-to-CL ratio. Note that mean body mass estimates are based on directly measured turtles and estimates based on regression (see methods in main text for details).

**Species**	**Max. *N****	**Habitat**	**Shell mass (g)**	**Cranial mass (g)**	**Total skeletal mass (g)**	**PL (mm)**	**CL (mm)**	**PL-to-CL ratio**	**Body mass (g)**
Claudius angustatus	1	Aquatic	19.60	3.00	30.50	63.20	101.00	0.626	174.20
Kinosternon abaxillare	6	Semiterrestrial	75.92	1.90	84.28	128.67	132.62	0.970	321.17
Kinosternon acutum	3	Semiterrestrial	41.09	0.88	43.99	96.62	100.97	0.957	123.73
Kinosternon albogulare	10	Semiterrestrial	158.29	2.73	170.25	141.55	148.00	0.956	586.54
Kinosternon baurii	32	Semiterrestrial	31.55	1.02	34.92	84.68	90.43	0.936	108.25
Kinosternon chimalhuaca	11	Semiterrestrial	51.52	2.21	60.24	107.04	123.13	0.873	272.18
Kinosternon creaseri	5	Semiterrestrial	46.97	1.63	51.40	105.26	112.92	0.933	181.00
Kinosternon cruentatum	13	Semiterrestrial	97.75	1.81	104.18	113.68	116.64	0.975	292.23
Kinosternon durangoense	4	Semiterrestrial	84.42	3.96	85.59	117.98	130.15	0.911	415.50
Kinosternon flavescens	62	Semiterrestrial	48.30	1.62	56.17	102.84	111.83	0.923	277.88
Kinosternon herrerai	7	Aquatic	70.26	4.00	84.18	115.86	139.81	0.831	409.00
Kinosternon hippocrepis	35	Semiterrestrial	27.34	0.82	30.46	83.06	87.97	0.943	161.94
Kinosternon hirtipes	69	Aquatic	66.61	2.37	74.01	115.04	129.87	0.887	333.48
Kinosternon integrum	45	Semiterrestrial	109.63	2.90	123.07	143.13	150.77	0.951	530.37
Kinosternon leucostomum	34	Semiterrestrial	87.32	2.15	93.54	114.30	123.90	0.923	292.95
Kinosternon mexicanum	1	Semiterrestrial	111.60	2.60	120.10	125.80	128.30	0.981	363.90
Kinosternon oaxacae	6	Semiterrestrial	85.56	3.18	98.62	130.04	147.52	0.885	409.90
Kinosternon scorpioides	6	Semiterrestrial	89.73	2.83	99.12	127.64	138.02	0.925	284.55

Kinosternon sonoriense	24	Semiterrestrial	66.11	1.93	67.43	115.70	125.75	0.919	323.02
Kinosternon steindachneri	22	Semiterrestrial	27.48	1.47	31.96	78.55	92.47	0.852	133.89
Kinosternon stejnegeri	9	Semiterrestrial	120.28	2.27	134.98	136.99	148.37	0.924	533.51
Kinosternon subrubrum	4	Semiterrestrial	27.48	0.60	30.20	87.77	94.83	0.926	171.80
Staurotypus salvinii	4	Aquatic	82.20	4.95	98.85	89.53	142.50	0.628	485.05
Staurotypus triporcatus	5	Aquatic	253.42	14.83	293.80	149.35	220.93	0.677	1385.83
Sternotherus carinatus	23	Aquatic	61.38	3.64	70.61	88.57	121.00	0.733	266.51
Sternotherus minor	48	Aquatic	31.10	2.73	37.05	74.82	98.74	0.757	161.27
Sternotherus odoratus	140	Aquatic	19.62	1.04	22.55	67.16	89.91	0.750	111.99

* = Maximum number of specimens sampled per species. Sample sizes per trait and sex may otherwise vary owing to incomplete sampling (see supplementary data file).
